# Clinical Significance of Intramural Metastasis as an Independent Prognostic Factor in Esophageal Squamous Cell Carcinoma

**DOI:** 10.1245/s10434-023-13464-w

**Published:** 2023-06-05

**Authors:** Yuki Ushimaru, Tomoki Makino, Koji Tanaka, Kotaro Yamashita, Takuro Saito, Kazuyoshi Yamamoto, Tsuyoshi Takahashi, Yukinori Kurokawa, Kiyokazu Nakajima, Eiichi Morii, Hidetoshi Eguchi, Yuichiro Doki

**Affiliations:** 1 Department of Gastroenterological Surgery, Osaka University Graduate School of Medicine, Osaka, Japan; 2 Department of Pathology, Osaka University Graduate School of Medicine, Osaka, Japan

## Abstract

**Background:**

Although intramural metastasis (IM) in esophageal cancer is considered a poor prognostic factor, there are only limited reports detailing its clinicopathologic characteristics and prognostic impact.

**Patients and Methods:**

We retrospectively included patients with esophageal squamous cell carcinoma (ESCC) with esophagectomy at our institution between 2010 and 2016. We compared patients with intramural metastases (IMs) (IM group) versus those without IMs (non-IM group) to clarify the clinical significance of intramural metastasis in ESCC.

**Results:**

A total of 23 (3.9%) out of all 597 patients were identified to have IM. The IMs were located on the cranial side in 13 (56.5%) and caudal side in 10 (43.5%) of the primary tumor, with two multiple cases. The IM group, compared with the non-IM group, was associated with higher percentage of cN-positive (91.3 versus 67.9%, *P* = 0.02), pN-positive (82.6 versus 55.9%, *P* = 0.04), and pM(lym)-positive (30.4 versus 12.5%, *P* = 0.02) cases. Five-year recurrence-free survival (RFS) was significantly worse in the IM group than the non-IM group (14.9 versus 55.0 %, *P* < 0.001). Multivariable analysis of recurrence-free survival identified pT (HR 1.74, 95% CI 1.36–2.23, *P* < 0.001), pN (HR 2.11, 95% CI 1.60–2.78, *P* < 0.001), histological classification (HR 1.68, 95% CI 1.21–2.35, *P* = 0.002), and pM(LYM) (HR 1.64, 95% CI 1.64–2.95, *P* < 0.001), along with presence of IM (HR 2.24, 95% CI 1.37–3.64, *P* < 0.001) to be independent prognostic factors. Lymphatic (65.2 versus 24.9%, *P* < 0.001) and hepatic (26.1 versus 6.8%, *P* = 0.005) recurrences were significantly more common in the IM group than in the non-IM group.

**Conclusions:**

IM was shown to be associated with dismal survival after surgery. A treatment strategy emphasizing more intensive systemic control should be considered for patients with ESCC with IM.

**Supplementary Information:**

The online version contains supplementary material available at 10.1245/s10434-023-13464-w.

Intramural metastasis (IM), defined as metastatic tumors from the primary tumor to the gastrointestinal tract via the intramural lymphatic system, is the mode of metastasis most likely to occur in esophageal, gastric, and colorectal cancers, especially esophageal squamous cell carcinoma (ESCC).^1^ IM in ESCC became widely known after it was reported by Watson et al. in 1933.^2^ IM of esophageal cancer is a unique metastatic pathway that differs from hematogenous and lymphatic metastasis and is an independent prognostic factor for esophageal cancer.^3^ When two or more unconnected cancer lesions are present in the esophagus, there are two possibilities: multiple primary sites and intramucosal metastases. However, the prognosis for numerous primary lesions is similar to that for a single tumor and is relatively favorable.^4,5^ On the other hand, patients with esophageal cancer with IM often have advanced lymph node metastasis and lymphatic invasion showing a poor prognosis.^6–11^ Therefore, IM was considered to have a significant impact on the decision of treatment strategy.^3^

The lamina propria of the esophageal mucosa contains a large number of densely distributed longitudinal lymphatic vessels. The lymphatic vessels in the submucosa are also abundant and form a lymphatic plexus, mainly distributed longitudinally, with a small portion distributed circumferentially around the esophagus. Accordingly, the longitudinal lymphatic vessels in the intramucosal and submucosal layers of the esophagus provide the anatomical basis for intramural metastasis. ^12^ In addition, the submucosal lymphatic drainage in the middle and lower esophagus might be similar with that in the gastric fundus; therefore, tumor cells may metastasize to the stomach through the submucosal lymphatic system, which could be the basis for intramural metastasis.^13^

In the 11th edition of the Japanese Esophageal Cancer Statute, esophageal cancer with IMs is recorded as IM1, and in particular, IMs in the gastric wall (recorded as “IM1-St”) are classified as organ metastasis (M1).^14^ However, the American Joint Committee on Cancer/Union for International Cancer Control (AJCC/UICC) tumor, nodes, and metastases (TNM) staging does not take this factor into account because ESCC is rare in Western countries, and cases of IMs are even rarer. Although several studies have reported the clinical significance of IM in preoperatively untreated ESCC,^6,7,9^ few studies have demonstrated the significance of IM now that multidisciplinary therapy has become the mainstay of treatment for advanced esophageal cancer. The purpose of this study is to clarify the clinical significance of IMs in ESCC.

## Patients and Methods

### Patients

We retrospectively collected data on 640 consecutive patients who underwent esophagectomy for esophageal squamous cell carcinoma between January 2010 and December 2016 in our single institute. Demographic characteristics, operative details, pathologic findings, perioperative treatment, and follow-up data for survival and recurrence were obtained from medical records, telephone interviews, and our institute database.

All patients were histologically diagnosed as having esophageal squamous cell carcinoma. Patients who underwent R1 or R2 resection, patients with gastric intramural metastasis, and patients who had esophageal cancer with histology other than squamous cell carcinoma were excluded from the analysis. In general, patients underwent esophagectomy with lymph node dissection according to Esophageal Cancer Practice guidelines.^15–17^ The longitudinal location of ESCC was identified according to the Japanese classification of esophageal cancer and divided into the following portions: upper thoracic esophagus (Ut), middle thoracic esophagus (Mt), and lower thoracic esophagus (Lt).^18^ TNM staging was determined on the basis of the 11th edition of the Japanese Classification of Esophageal Cancer.^18^

### Preoperative and Surgical Treatment

Our indication for neoadjuvant chemotherapy (NAC) was based on the TNM classification, as follows: cT1-3N1-3 was an absolute indication, and either cT3N0 or cT4Nany was a relative indication, except when massive infiltration to the bronchus or aorta had occurred.^19–21^ Patients with cT1-2N0 underwent surgery without NAC. Patients received the following NAC regimens: docetaxel, cisplatin plus 5-fluorouracil (DCF) chemotherapy, which was made up of docetaxel 70 mg/m2 (day 1), cisplatin 70 mg/m2 (day 1), and 5-FU 700 mg/m2 (days 1–5).^19,22–25^ Our standard surgical treatment consisted of a subtotal esophagectomy with two or three field lymphadenectomies, reconstruction of the gastric tube via the retrosternal or posterior mediastinal route, and anastomosis in the cervical area from the cervical incision, as described previously.^26–28^ After surgery, the patients were surveyed every 3 months by physical examination and measurement of serum tumor markers, every 6 months by computed tomography (CT) scan and abdominal ultrasonography, and every year by endoscopy until tumor recurrence was evident. Patients with tumor recurrence received chemotherapy or chemoradiotherapy as long as their systemic condition permitted.

### Diagnosis and Definition of Intramural Metastasis

Intramural metastasis was defined as a metastatic lesion from a primary tumor of the thoracic esophagus meeting the following macroscopic and histologic criteria: (1) clearly separated from the primary tumor; (2) located in the esophageal wall; (3) having a gross appearance of a submucosal tumor without intraepithelial cancer extension; (4) having the same histologic type as the primary tumor in the cases with the histological examination; and (5) lacking any evidence of intravascular growth. These criteria discriminated IMs from multiple primary tumors in the esophagus or stomach and from intravascular tumor emboli around the primary tumor. In the case that endoscopic biopsy did not lead to a diagnosis, the IM diagnosis was based on comprehensive findings, including CT, positron emission tomography (PET)-CT, and postoperative pathology. The absence or presence of IM was assessed macroscopically and histologically on the resected specimens and preoperative endoscopic examinations. Cases diagnosed with IMs by any method were classified into the IM group, including complete response (CR) cases on postoperative pathology.

Patients with IM were defined as the IM group (*n* = 23, 3.9%) and those without IM as the non-IM group (*n* = 574, 96.1%), and background factors and long-term prognosis were examined.

### Statistical Analysis

Overall survival (OS) was defined as the period from the date of surgery to the date of death from any cause. Recurrence-free survival (RFS) was defined as the time between the date of surgery and date of the first recurrence (local, regional, or distant metastasis) or death, whichever occurred first, while the patients who died from other causes were analyzed by censoring the data. Survival was estimated using the Kaplan–Meier method and compared using the log-rank test. The Cox proportional hazards model was used to determine the hazard ratio (HR) of variables on overall or recurrence-free survival in univariate and multivariable analysis to adjust for age, gender, pathological TNM status, tumor location, histological classification, and presence of IM. We compared the clinicopathological characteristics of the IM and non-IM groups using the chi-squared test and Fisher’s exact test for categorical variables, and Student’s t-test for continuous variables. *P* < 0.05 was considered to indicate a statistically significant difference. All statistical analyses were performed using JMP PRO software (JMP version 16.1.0, SAS Institute, Cary, NC).

## Results

### Patient Characteristics

**Fig. 1:**
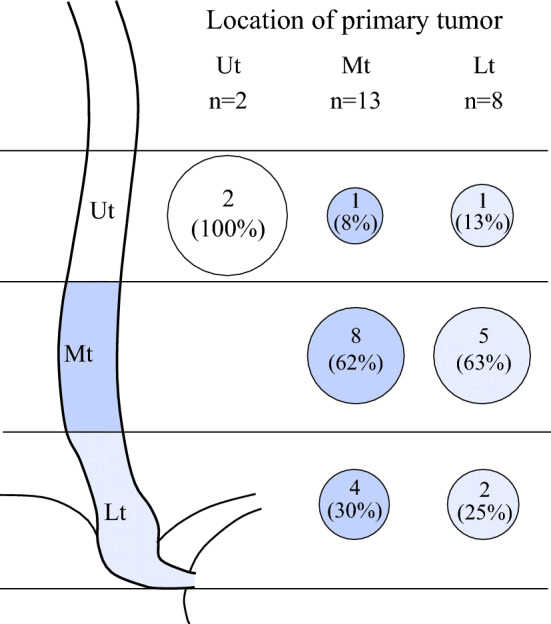
Distribution of intramural metastasis according to the location of the primary tumor

Of the 597 patients who underwent radical esophageal cancer resection, 23 (3.9%) had IMs. The details of IM are presented in Table 1 and Fig. 1. The IMs were located on the cranial side in 13 (56.5%) and the caudal side in 10 (43.5%) of the primary tumor, with a median distance of 1 cm (range: −11–18 cm) away. In cases with the primary lesion in Ut, all IMs were located near the primary lesion; in cases with the primary lesion in Mt, IMs were distributed on both the cranial and caudal sides, but most frequently at Mt (*n* = 8, 62%). In contrast, in cases with the primary lesion in Lt, IMs were distributed on the cranial side, mainly at Mt (*n* = 5, 63%). In total, 21 (91.3%) cases had solitary IM lesions, while only 2 (8.7%) cases showed multiple lesions, and 18 patients (78.3%) were diagnosed by endoscopic biopsy before treatment. The remaining five cases (22%) were diagnosed comprehensively by multifaceted examinations, including endoscopy, CT scan, PET-CT, and postoperative pathology; among these cases, IM was reconfirmed in postoperative pathology of three cases. Among 21 cases (91.3%) who received NAC, 18 cases (78.3%) showed complete response (CR) and 3 (13.0%) partial response (PR) as NAC response of IMs. Cases diagnosed with IMs in any way were classified into the IM group, even if they became CR after NAC.

**Table 1:** Details of intramural metastasis

	IM group (*n* = 23)
*Numbers of IMs*	
1	21 (93%)
2 or more	2 (7%)
*Location of IMs*	
Cranial side of primary tumor	13 (57%)
Caudal side of primary tumor	10 (43%)
Distance from intramural metastasis to primary tumor (cm)*	
All the tumor	1 (−11 to 18)
Ut	2 (1 to 3)
Mt	−1 (−11 to 4)
Lt	4 (−6 to 18)
*Diagnostic approach of IMs*	
Pretreatment endoscopic biopsy diagnosis	18 (78.3%)
Comprehensive imaging diagnosis	5 (21.7%)
*Clinical response of IMs to neoadjuvant chemotherapy*	
Complete response	18 (78.3%)
Partial response	3 (13.0%)
No preoperative treatment	2 (8.7%)

^*^Distance from intramural metastasis to the primary lesion is defined as plus on the cranial side and minus on the caudal side

Although there were more cN-positive patients in the IM group compared with the non-IM group (91.3 versus 67.9%, *P* = 0.02), no differences were observed in other background factors such as age and gender, tumor localization, degree of progression, multiple organ metastasis, and presence of chemotherapy (Table 2). In addition, the IM group was associated with the more advanced pN (pN1-3; 82.6 versus 55.9%, *P* = 0.04) and pM (LYM) (pM1; 30.4 versus 12.5%, *P* = 0.02) compared with the non-IM group (Table 3).

**Table 2:** Patients’ characteristics

	IM group (*n* = 23)	Non-IM group (*n* = 574)	*P*-value
Age (year)*	68 (49–83)	67 (16–90)	0.73
Gender			0.76
Male	21 (91%)	494 (86%)	
Female	2 (9%)	80 (14%)	
Tumor location			0.12
Ut	2 (9%)	155 (27%)	
Mt	13 (57%)	255 (44%)	
Lt	8 (35%)	164 (29%)	
Histological differentiation (squamous cell carcinoma)			0.36
Well	3 (13%)	144 (25%)	
Moderately	17 (74%)	373 (65%)	
Poorly	3 (13%)	57 (10%)	
Clinical T status			0.93
T1–2	7 (30%)	180 (31%)	
T3–4	16 (70%)	394 (69%)	
Clinical N status			0.02
N0	2 (9%)	184 (32%)	
N1–3	21 (91%)	390 (68%)	
Clinical M status			0.55
M0	19 (83%)	493 (86%)	
M1**	4 (17%)	81 (14%)	
Clinical stage			0.26
I	3 (13.0%)	129 (22.5%)	
II–IV	20 (87.0%)	445 (77.5%)	
Neoadjuvant chemotherapy			0.66
Presence	21 (91%)	486 (84%)	
Absence	2 (9%)	88 (16%)	

Values are presented as median (range) (*) or number (%). *P* = 0.05 was considered statistically significant. **Supraclavicular lymph node metastasis

**Table 3:** Pathological findings

	IM group (*n* = 23)	Non-IM group (*n* = 574)	*P*-value
Pathological T status			0.19
T0–2	10 (43.5%)	330 (57.5%)	
T3–4	13 (56.5%)	244 (42.5%)	
Pathological N status			0.008
N0	4 (17.4%)	253 (44.1%)	
N1–3	19 (82.6%)	321 (55.9%)	
Pathological M status			0.029
M0	16 (69.6%)	498 (87%)	
M1	7 (30.4%)	76 (13%)	
Pathological stage			0.34
I	6 (26.1%)	204 (35.5%)	
II–IV	17 (73.9%)	370 (64.5%)	
Lymphatic invasion			0.28
Positive	16 (70%)	336 (59%)	
Negative	7 (30%)	238 (41%)	
Venous invasion			0.96
Positive	7 (30%)	172 (30%)	
Negative	16 (70%)	402 (70%)	

Values are presented as numbers (%). *P* = 0.05 was considered statistically significant

### Long-Term Outcomes

**Fig. 2:**
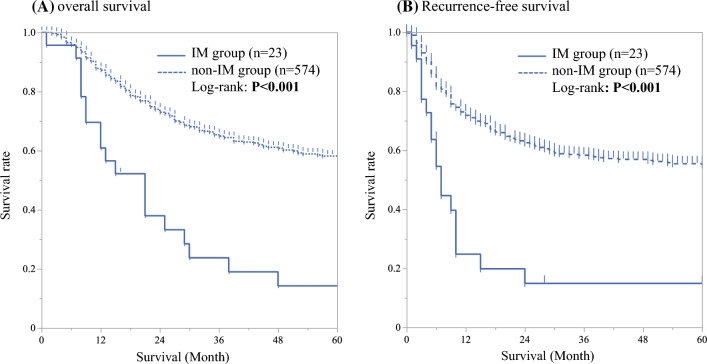
Recurrence-free survival curve and overall survival curve of the IM group versus non-IM group

In all cases, 5-year OS (14.2 versus 58.2%, *P* < 0.001) and RFS (8.7 versus 49.6 %, *P* < 0.001) were significantly worse in the IM group compared with the non-IM group (Fig. 2). In multivariable analysis of OS, presence of IM [HR 2.02, 95% confidence intervals (95% CI) 1.25–3.28, *P* = 0.004] along with gender (HR 1.76, 95% CI 1.09–2.82, *P* = 0.02), pT (HR 2.21, 95% CI 1.68–2.91, *P* < 0.001), pN (HR 1.96, 95% CI 1.43–2.67, *P* < 0.001), histological differentiation (HR 1.68, 95% CI 1.19–2.38, *P* = 0.003), pM (LYM) (HR 1.40, 95% CI 1.00–1.96, *P* = 0.048), and tumor location (HR 1.46, 95% CI 1.09–2.38, *P* < 0.001) were found to be independent prognostic factors (Supplementary Table1). Similarly, multivariable analysis of RFS identified pT (HR 1.74, 95% CI 1.36–2.23, *P* < 0.001), pN (HR 2.11, 95% CI 1.60–2.78, *P* < 0.001), histological differentiation (HR 1.68, 95% CI 1.21–2.35, *P* = 0.002), pM (LYM) (HR 1.64, 95% CI 1.64–2.95, *P* < 0.001), and presence of IM (HR 2.24, 95% CI 1.37–3.64, *P* < 0.001) to be independent prognostic factors (Table 4).

**Table 4:** Univariate/multivariable of recurrence-free survival

	Univariate analysis		Multivariable analysis	
	Hazard ratio (95% confidence interval)	*P*-value	Hazard ratio (95% confidence interval)	*P*-value
Age (year)	0.991	0.17		
	(0.978–1.004)			
Gender	1.356	0.1		
Male/female	(0.943–1.949)			
Pathological T status	2.41	< 0.001	1.741	< 0.001
T3–4/T0–2	(1.908–3.045)		(1.360–2.230)	
Pathological N status	2.882	< 0.001	2.107	< 0.001
pN1–3/pN0	(2.221–3.738)		(1.597–2.780)	
Tumor location	1.099	0.48		
Ut / Mt, Lt	(0.848–1.425)			
Histological differentiation	1.781	0.001	1.684	0.002
Poorly/well, moderately	(1.278–2.481)		(1.208–2.347)	
Pathological M status (LYM)	3.438	< 0.001	1.642	< 0.001
M1*/M0	(2.606–4.536)		(1.642–2.952)	
Intramural metastasis	3.231	< 0.001	2.236	0.001
IM1/IM0	(1.995–5.232)		(1.374–3.638)	

^*^Supraclavicular lymph node metastasis

### Recurrence Pattern

Overall postoperative recurrence rates were significantly higher in the IM group compared with the non-IM group (78.2 versus 37.3%, *P* < 0.001) (Table 5). In terms of pattern of disease recurrence, lymphatic (65.2 versus 24.9%, *P* < 0.001) and liver metastasis (26.1 versus 6.8%, *P* = 0.005) were significantly more common in the IM group than the non-IM group, while other types of disease recurrence did not significantly differ between the two groups.

**Table 5:** Pattern of disease recurrence

		IM group (*n* = 23)	Non-IM group (*n* = 574)	*P*-value
All the recurrence	Yes	18 (78%)	212 (37%)	< 0.001
	No	5 (22%)	362 (63%)	
Local	Yes	1 (4%)	19 (3%)	
	No	22 (96%)	555 (97%)	
Lymph node	Yes	15 (65%)	143 (25%)	
	No	8 (35%)	431 (75%)	
Peritoneal metastasis	Yes	3 (13%)	33 (6%)	
	No	20 (87%)	541 (94%)	
Lung	Yes	2 (9%)	47 (8%)	
	No	21 (91%)	527 (92%)	
Liver	Yes	6 (26%)	39 (7%)	
	No	17 (74%)	535 (93%)	
Bone	Yes	2 (9%)	17 (3%)	
	No	21 (91%)	557 (97%)	
Brain	Yes	1 (4%)	11 (2%)	
	No	22 (96%)	563 (98%)	
Others	Yes	0 (0%)	4 (1%)	
	No	23 (00%)	570 (99%)	

Values are presented as numbers (%). *P* = 0.05 was considered statistically significant

## Discussion

In this study, IM, mostly solitary lesion, was identified in 3.9% of all ESCC patients. The middle thoracic esophagus was the most common lesion of IM with a median of 3 cm away from the primary tumor. The IM group significantly correlated with more advanced cN, pN, and pM compared with the non-IM group. Notably, the IM group was associated with poorer overall and recurrence-free survival, and the presence of IM was found to be an independent prognostic factor in multivariable analysis. The IM group more often developed lymphatic/liver recurrence after surgery, suggesting the necessity for further systemic control to improve prognosis.

In general, patients with a large number of lymph node metastases are known to show shorter postoperative survival and higher risk of disease recurrence, particularly distant metastasis.^3,29–31^ Therefore, lymph node status as histologically assessed in the resected specimen is recognized as the most crucial factor affecting prognosis of patients with ESCC. The lymphatic drainage system of the esophagus is highly complex due to an abundant lymph capillary network in the deep basement membrane, in both the lamina propria and mucosal muscularis propria. There are two ways of lymphatic spreading: transverse through the esophageal wall and longitudinal shift. Longitudinal spreading is often abundant in the esophagus.^32,33^ This large submucosal lymph node network often leads to longitudinal lymph node metastasis; IM, the focus of this study, is generated via the submucosal lymphatic system. In fact, patients with IM have a greater tumor burden (i.e., more advanced N and M stage) than those without IM. On the other hand, the fewer occurrences of IM in Ut ESCC may indicate a difference in lymphatic flow compared with Mt/Lt tumors, which may require further investigation.

In the present study, the rate of intramural metastases was lower than previously reported. In patients with ESCC, IMs have been reported to range from 5.0 to 16.6% and have been suggested to be associated with large tumor size and T and N stage progression.^6–11^ In the present study, the majority of IM lesions were single (single: 93%, multi: 7%) and located in the vicinity of the primary lesion in the Ut/Mt region. On the contrary, IM in the case of Lt primary lesion often spread to Mt. In our study, the frequency of IMs in ESCC was 3.9%, lower than in previous reports. Since approximately 90% of the patients received preoperative chemotherapy, the IM may have disappeared in patients who responded well to prior treatment, implying the possibility of underestimation regarding IM frequency.

A standard treatment strategy for patients with ESCC with IM has not yet been established. In Japan, NAC with docetaxel and DCF followed by surgery became the standard treatment for patients with resectable advanced esophageal cancer following JCOG 1109.^34^ In other studies, this triplet chemotherapy, DCF, has shown potential as an optional NAC treatment for locally advanced ESCC.^22,35,36^ However, it has not been proven yet whether NAC improves survival in patients with IM.^37,38^ In addition, patients who do not respond to preoperative treatment have a high recurrence rate and need time to improve their general condition after esophageal cancer surgery, making intense postoperative chemotherapy impossible. In CheckMate 577, 1 year of postoperative nivolumab significantly prolonged DFS in patients who did not achieve pathological complete response after preoperative chemoradiation.^39^ However, there is currently no evidence for immune checkpoint inhibitor (ICI) after preoperative DCF followed by surgery because preoperative treatment is defined as chemoradiotherapy in CheckMate 577. There is even less evidence for postoperative ICI for ESCC with IMs. Future data on postoperative ICI after preoperative DCF are expected to emerge from multicenter, observational, and translational research studies using liquid biopsies such as circulating tumor DNA and immunological biomarkers.

There are several limitations to this study. First, this is a retrospective observational study conducted at a single institution over a relatively long period. Second, the small number of patients with IM may have limited statistical power. Regarding multivariable analyses of OS and RFS, the number of variables analyzed was relatively high for the number of actual events in the present study, and therefore, we should consider as a limitations the potential of overfitting in the models although the 95% CI was relatively narrow. Third, since about 90% of patients received NAC, IM frequency is likely to be underestimated in the present study as previously described. Furthermore, NAC was introduced at our institution before the results of JCOG 9907.^40^ Since all included patients were treated after 2010, it is unlikely that the presence or absence of NAC could have affected the survival results. A prospective multicenter cohort study that can exclude the impact of NAC is needed to validate our findings.

In conclusion, the present study demonstrated that IM was identified in 3.9% of patients with ESCC who underwent surgery. The presence of IM was associated with the status of lymph node metastases, including supraclavicular lymph nodes, and identified to be an independent prognostic factor for both recurrence-free and overall survival. Patients with IM more often developed lymph node and liver recurrences after surgery, suggesting that future studies should focus on the benefit of additional therapy in this subgroup of patients with worse prognoses.

## Supplementary Information

Below is the link to the electronic supplementary material.

Supplementary file1 (DOCX 13 kb)
