# Supplementary material for: Clinical Significance of Intramural Metastasis as an Independent Prognostic Factor in Esophageal Squamous Cell Carcinoma
Source: Ann Surg Oncol. 2023 Jun 5;30(8):5195–202. doi: 10.1245/s10434-023-13464-w (PMC10319648; doi:10.1245/s10434-023-13464-w)
Supplement: Supplementary file 1 — Supplementary file1 (DOCX 13 kb) [file 10434_2023_13464_MOESM1_ESM.docx]

**SUPPLEMENTARY TABLE 1** *Predictive factors for overall survival*

|  | Univariate analysis | |  | Multivariable analysis | |
| --- | --- | --- | --- | --- | --- |
|  | Hazard ratio  (95% confidence interval) | *P*-value |  | Hazard ratio  (95% confidence interval) | *P*-value |
| Age (year) | 2.272  (0.734–7.245) | 0.16 |  |  |  |
| Gender  Male/female | 1.886  (1.180–3.016) | 0.004 |  | 1.761  (1.099–2.822) | 0.019 |
| Pathological T status  T3–4/under T2 | 2.735  (2.102–3.559) | < 0.001 |  | 2.213  (1.683–2.911) | < 0.001 |
| Pathological N status  pN1–3/pN0 | 2.669  (1.997–3.569) | < 0.001 |  | 1.955  (1.430–2.674) | < 0.001 |
| Tumor location  Ut/Mt, Lt | 1.360  (1.008–1.835) | 0.039 |  | 1.464  (1.081–1.983) | 0.014 |
| Histological classification  Poor/well–moderate | 1.879  (1.332–2.652) | 0.001 |  | 1.682  (1.188–2.384) | 0.003 |
| Pathological M status (LYM)  M1*/M0 | 2.260  (1.695–3.095) | < 0.001 |  | 1.400  (1.002–1.957) | 0.048 |
| Intramural metastasis  IM1/IM0 | 3.220  (2.011–5.157) | < 0.001 |  | 2.021  (1.246–3.279) | 0.004 |

*Supraclavicular lymph node metastasis
